# Facile reduction of graphene oxide suspensions and films using glass wafers

**DOI:** 10.1038/s41598-018-32488-x

**Published:** 2018-09-20

**Authors:** Maxim K. Rabchinskii, Arthur T. Dideikin, Demid A. Kirilenko, Marina V. Baidakova, Vladimir V. Shnitov, Friedrich Roth, Sergei V. Konyakhin, Nadezhda A. Besedina, Sergei I. Pavlov, Roman A. Kuricyn, Natalie M. Lebedeva, Pavel N. Brunkov, Alexander Ya. Vul’

**Affiliations:** 1 Ioffe Institute, 26 Politekhnicheskaya, Saint-Petersburg, 194021 Russia; 2 ITMO University, 49 Kronverksky Pr., Saint-Petersburg, 197101 Russia; 3 TU Bergakademie Freiberg, Institute of Experimental Physics, Freiberg, D-09599 Germany; 4 St. Petersburg Academic University, St. Petersburg, 194021 Russia; 5 Institute Pascal, PHOTON-N2, University Clermont Auvergne, CNRS, 63178 Aubiere Cedex, France

## Abstract

This paper reports a facile and green method for conversion of graphene oxide (GO) into graphene by low-temperature heating (80 °C) in the presence of a glass wafer. Compared to conventional GO chemical reduction methods, the presented approach is easy-scalable, operationally simple, and based on the use of a non-toxic recyclable deoxygenation agent. The efficiency of the proposed method is further expanded by the fact that it can be applied for reducing both GO suspensions and large-scale thin films formed on various substrates prior to the reduction process. The quality of the obtained reduced graphene oxide (rGO) strongly depends on the type of the used glass wafer, and, particularly, magnesium silicate glass can provide rGO with the C/O ratio of 7.4 and conductivity of up to 33000 S*cm^−1^. Based on the data obtained, we have suggested a mechanism of the observed reduction process in terms of the hydrolysis of the glass wafer with subsequent interaction of the leached alkali and alkali earth cations and silicate anions with graphene oxide, resulting in elimination of the oxygen-containing groups from the latter one. The proposed approach can be efficiently used for low-cost bulk-quantity production of graphene and graphene-based materials for a wide field of applications.

## Introduction

Graphene continues to inspire interest in various fields of science due to its outstanding physical and chemical properties^1–3^, even though intensive studies devoted to this unique nanocarbon material^4,5^ have been carried out during the last ten years. It seems to have a wide field of applications in different technologies, including fabrication of transparent electrodes, supercapasitors and nanoelectronic devices, gas sensing and catalysis, biochemistry and microbiology^6–9^.

However, the preparation of graphene in large scales remains to be a challenging task. Several strategies have been developed to overcome this challenge, such as epitaxial growth of graphene on silicon carbide (SiC)^10^, growth of graphene on the surface of transition metals by chemical vapor deposition (CVD)^11^, and reduction of graphene oxide (GO)^12^. Among these methods, the reduction proved to be an effective approach to produce graphene with an optimal quality at relatively low cost^13,14^.

Numerous approaches are applied to achieve conversion of graphene oxide to graphene, for instance, high-temperature annealing in a reducing enviroment^15^, ultraviolet^16^ and laser^17^ irradiation of GO films, electrochemical^18^ and chemical^19^ treatment of graphene oxide suspensions and films. Compared to other techniques, the chemical reduction of GO offers great ease of large-scale production of rGO in various forms such as suspensions, rGO paper or thin films on various substrates.

Various chemicals, namely, hydrazine monohydrate^20^, dimetilhydrazine^21^, hydroquinone^22^ or sodium borohydride^23^ are typically employed in chemical reduction of GO. However, these reducing agents are highly toxic, unrecyclable, highly unstable, or generate hazardous by-products. Moreover, strong acidic or alkaline conditions are commonly required in these reduction processes thus limiting their applicability for preparing graphene-containing polymer composites^24^ when the reduction process must be carried out simultaneously with introducing graphene into the polymer matrix.

On the other hand, the use of mild reducing agents, such as green molecules presented by different types of sugars (glucose or sucrose)^25^ or L-ascorbic acid^26^, results in a drastic increase in time required for the reduction of GO, and usually takes place in hot solutions. Furthermore, effective reduction of GO by these chemicals commonly requires addition of ammonia that limits the subsequent use of the obtained rGO suspensions. As such, the development of new chemical methods able to provide rapid and efficient reduction of graphene oxide under mild experimental conditions with the use of a reusable deoxygenation agent is of a high interest nowadays.

In this paper we report a new facile method to convert GO to graphene under low-temperature heating by using sodium, alkali-barium and magnesium silicate glass wafers as reducing agents. The method can be applied for both GO aqueous suspensions and large-scale GO films formed on various substrates prior to reducing. A possible model of the GO reduction using different glass wafers is proposed. Overall, we demonstrate that reduction of GO via low-temperature heating in the presence of glass wafers appears to be a green, efficient and easily scalable method that is based on the use of recyclable non-toxic reducing agent and can be effectively employed for low-cost bulk-quantity production of graphene and graphene-based derivatives.

## Results

### Optical images and UV-Vis spectra

**Figure 1:**
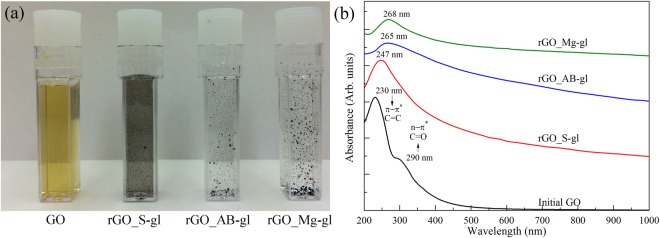
(**a**) Optical photographs of the initial graphene oxide aqueous suspension of 0.01 wt% (GO) and GO aqueous suspensions of 0.01 wt%, reduced using sodium silicate glass (rGO_S-gl), alkali-barium silicate glass (rGO_AB-gl), magnesium silicate glass (rGO_Mg-gl). (**b**) The UV-Vis spectra of the initial GO and rGOs obtained using various glass wafers as reducing agents. The spectra are vertically offset for clarity.

Figure 1a presents photographs of the initial GO aqueous suspension and GO suspensions (GO) after heating them at 80 °C in the presence of sodium silicate glass (rGO_S-gl), alkali-barium silicate glass (rGO_AB-gl) and magnesium silicate glass (rGO_Mg-gl) wafers. The suspension color change from yellow to black is an obvious visible characteristic of the successful conversion of GO into graphene^27^. The removal of the hydrophilic functional groups is further evidenced by the aggregation of rGO sheets as a result of π-π stacking interactions. The rate of the aggregation rises from the rGO_S-gl to rGO_Mg-gl sample. This fact suggests more complete elimination of oxygen-containing functional groups and larger areas of the graphene network in the latter one.

Figure 1b shows the UV-Vis spectra of GO and rGO samples. The initial GO exhibits two distinctive features, the main absorption peak at 230 nm due to π-π* transitions of C=C bonds and a broadband absorption shoulder centered at 300 nm. The latter one is commonly attributed to n-π* transitions of C=O bonds of carbonyl and carboxyl groups^28^. However, it also can arise from optical transitions between π and π* states in the nanometer-size *sp*^2^ clusters remained in the structure of GO after its oxidation^29^. Upon the reduction, the 230 nm absorption peak progressively shifts towards higher wavelengths, and overall absorption in the range up to near-infrared (NIR) region rises significantly due to the restoration of *sp*^2^-conjugated graphene network. As seen, the UV-Vis spectra of rGO_Mg-gl and rGO_AB-gl are almost similar with the peak of the π-π* transition lying at 265–268 nm, which is a characteristic feature of the high-degree GO reduction^30^. At the same time, the position of the main absorption peak (at 247 nm) and highly non-linear character of the absorption in the visible and NIR regions in the rGO_S-gl spectrum signifies incomplete elimination of the oxygen functionalities in the case of using sodium silicate glass as a reducing agent.

### FTIR and XPS spectra

**Figure 2:**
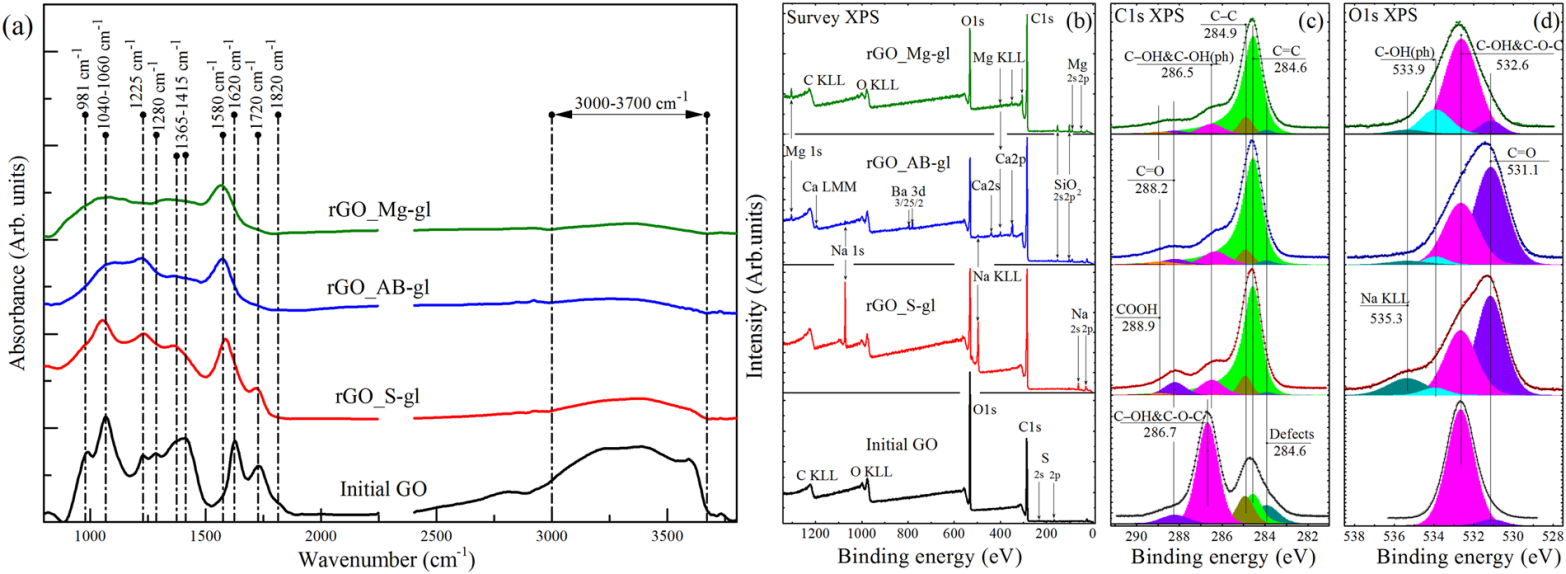
(**a**) FTIR spectra of the initial graphene oxide and GO reduced by using glass wafers. The spectra are vertically offset for clarity. (**b**) Survey (**a**) and high-resolution C1s (**b**) and O1s (**c**) XPS spectra of the initial GO and obtained rGOs. For clarity, C 1 s and O 1 s spectra and their fits are shown after Shirley background subtraction and vertically offset from the fitting components. The C 1s spectra were fitted by a set of one asymmetric Doniach-Sunjic function (DS) and five symmetric Gaussian−Lorentzian product functions (Gaussian by 70% and Lorentzian by 30%) (GL(30)), while the O 1s spectra were fitted by only the GL(30) functions whose number varied from 3 to 5.

Figure 2a presents the initial GO IR spectrum that exhibits a number of characteristic absorption bands related to the oxygen functionalities and interlayer water^31,32^. Particularly, the broadband absorption feature at 3000–3700 cm^−1^ originating from the set of overlapping bands of O-H stretching in water molecules, hydroxyls and carboxyls is observed. Additionally, distinguishable bands at 1720 cm^−1^, 1620 cm^−1^, 1415 cm^−1^, 1225 cm^−1^ and 1040 cm^−1^ are presented. These absorption lines correspond to the stretching and bending vibrations of the carbonyl/carboxyl groups, water molecules, basal-plane hydroxyls, epoxides and edge-located hydroxyls, respectively. The distinguishable features at 980 cm^−1^ and 1280 cm^−1^ are related to the presence of the five-membered ring lactols^32^ and ethers^33^.

The emergence of the prominent absorption band at 1580 cm^−1^ that corresponds to C=C vibrations within the recuperated conjugated aromatic structure and vanishing of the absorption bands at 3000–3700 cm^−1^ indicates successful deoxygenation of the rGOs. However, the IR spectrum of the rGO-Sgl sample still exhibits noticeable absorption bands of epoxides (1225 cm^−1^), edge-located hydroxyls (1040 cm^−1^) and carboxyls/carbonyls (1720 cm^−1^). This suggests retention of some amount these functionalities after the reduction. At the same time, the IR spectra for both rGO_AB-gl and rGO_Mg-gl samples demonstrate nearly complete elimination of all oxygen-containing functionalities. The only absorption feature that can be distinguished is related to edge-located hydroxyl groups (phenols). The preservation of these groups is due to their high resistivity to elimination^34^.

Figure 2b–d show the survey, high-resolution C 1 s and high-resolution O 1 s core level XPS spectra of the samples, respectively. The presence of prominent peaks of Na 1 s and Na KLL in the rGO_S-gl survey spectrum (Fig. 2b) demonstrates that a certain amount of Na (~4.9 at.%,) retains in the structure of this sample. The observed preservation of sodium in such concentrations can be understood in terms of substitution of the hydrogen ion in the residual hydroxyl and carboxyl groups with a sodium cation. At the same time, the analysis of the survey spectra of rGO_AB-gl and rGO_Mg-gl indicates that concentration of residual alkaline-earth metals in these samples is considerably low, appearing to be less than 0.8 at % and 0.3 at%, respectively.

In the C1s XPS spectra (Fig. 2c), six distinct peaks can be discerned. The peak at 283.9 eV is attributed to carbons that are the nearest neighbors of graphene vacancy defects^35^ (peak C-V). The peaks 284.6 eV and 284.9 eV are related to *sp*^2^-bonded carbons of perfect graphene lattice (peak C=C) and to carbon atoms being partially *sp*^3^-hybridized due to strong graphene network distortion caused by attachment of oxygen-containing groups (peak C-C), respectively^36^. Note that the C=C peak is asymmetric due to the natural asymmetry inherent for C1s XPS spectra of highly sp2-conjugated graphene-like structure observed in highly reduced GO films^37,38^. Other three peaks located at 286.7 eV, 288.2 eV and 288.9 eV correspond to hydroxyl and epoxide groups (C-OH and C-O-C), carbonyl groups (C=O) and carboxyl groups (COOH), respectively^36,39^. Three main O1s components (Fig. 2d) positioned at binding energies of 531.0, 532.5 and 533.6 eV are assigned, respectively, to the C=O bonds, C-O bonds within the basal plane groups (C-OH and C-O-C) and C-O bonds within phenols (C-OH(ph)) and carboxyls (O=(C-OH))^34^. Table 1 represents the results obtained by quantitative analysis of the deconvoluted C1s XPS spectra. High content of the oxygen-containing functional groups and low calculated C/O ratio give a hint that the initial GO is highly oxidized. After the reduction, the intensities of the C 1s peaks related to the oxidized groups decrease significantly, which is accompanied by a significant rise in the C/O ratios determined to be 4.22, 5.3 and 7.41 for rGO_S-gl, rGO_AB-gl and rGO_Mg-gl, respectively. These values are very close to those of rGO prepared by chemical reduction using common reducing agents, namely, hydrazine, benzylamine, various alcohols and sodium borohydride^19,40^.

**Table 1:** The C/O ratios and relative concentrations of functional groups determined by deconvolution of C1s XPS spectra for rGOs obtained using sodium silicate, alkali-barium silicate and magnesium silicate glasses.

Component	Defects	C=C	C-C	C-OH & C-O-C $({{\boldsymbol{D}}}_{\mathrm{GO}{\boldsymbol{/}}\mathrm{rGO}}^{{\boldsymbol{BasalPlane}}})$	>C=O $({{\boldsymbol{D}}}_{\mathrm{GO}{\boldsymbol{/}}\mathrm{rGO}}^{C{\boldsymbol{=}}O})$	O=C-OH $({{\boldsymbol{D}}}_{\mathrm{GO}{\boldsymbol{/}}\mathrm{rGO}}^{{\boldsymbol{COOH}}})$	C/O Ratio
Binding Energy (eV)	283.9	284.6	284.9	286.7	288.2	288.9	
GO	0.109	0.182	0.115	0.532	0.042	0.02	0.68
rGO_S-gl	0.010	0.729	0.058	0.098	0.077	0.028	4.22
rGO_AB-gl	0.016	0.768	0.063	0.095	0.039	0.018	5.30
rGO_Mg-gl	0.016	0.793	0.073	0.075	0.025	0.018	7.41

Beside the difference in the C/O ratio, the rGOs obtained using different glass wafers also exhibit distinct compositions of residual functionalities. The rGO_S-gl sample is characterized by the presence of a high number of carbonyl groups, which is indicated by the prominent 288.2-eV peak in the C 1 s spectrum and domination of the C=O component in the O 1 s spectrum. This suggests that reduction of GO by using sodium silicate glass lead to formation of new carbonyls. At the same time, according to the quantitative analysis of the deconvoluted C1s XPS the content of carbonyls in rGO_AB-gl is nearly the same as in GO, suggesting absence of their elimination during the treatment. This is also indicated by the relatively high intensity of the C=O peak in the rGO_AB-gl O 1 s spectrum, which significantly differs from the rGO_Mg-gl one. The observed difference in the O 1s spectra of these samples is also related to the higher concentration of retained alkaline earth carbonates formed during the reduction. This is evidenced by a higher content of the retained Ca, Ba, and Mg in rGO_AB-gl sample in comparison to the amount of the residual Mg in rGO_Mg-gl as can be seen from the survey XPS spectra.

### XRD patterns, Raman spectra and TEM images

**Figure 3:**
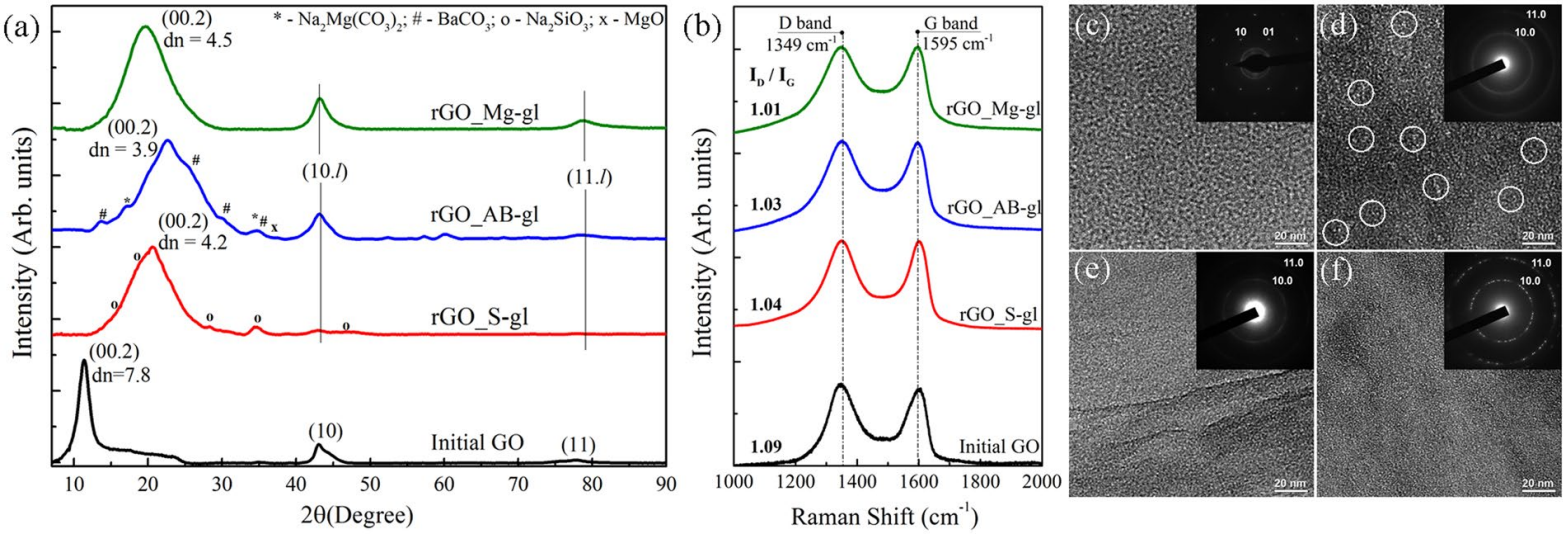
(**a**) XRD patterns of the initial GO and rGO samples. The d-value is given in Å. The (10.l) and (11.l) indicate diffraction reflections corresponding to the superposition of crystalline reflections of type (hk.l) and two-dimensional lattice reflections of type (hk). The reflection peaks of metal-containing contaminants are marked using the following PDF data: *-Na2Mg(CO3)2 (PDF No.00-024-1227); #-BaCO3 (PDF No.00-002-0364); х-MgO (PDF No.01-077-2906), o-Na2SiO3 (PDF No. 00-016-0818). (**b**) Raman spectra of the GO and rGO samples recorded using a 532-nm laser. TEM images and selected area diffraction (SAED) patterns (in the insets) of the (**c**) initial GO, (**d**) rGO_S-gl, (**e**) rGO_AB-gl and (**f**) rGO_Mg-gl samples. White circles denote nanoscale holes formed during reduction.

Figure 3a shows the diffraction pattern of the initial GO that exhibits a narrow peak at 2θ = 11.3° that corresponds to diffraction reflection from the (00.2) planes with the basal spacing (d_basal_) of 7.8 Å. This value is consistent with the published data^41^. Additionally, the less intense broadened peaks at 2θ = 43° and 2θ = 77.7° characteristic of the (10) and (11) reflections can be observed. These peaks arise from the 2D diffraction from the planar carbon network of GO flakes. The intensity ratio between the crystalline reflections of type (00.l) and lattice reflections of type (hk) indicates that the GO flakes have a lamellar structure, and their lateral size is larger than several micrometers^42^. Further analysis of the SEM images of arrays of GO flakes on the silicon wafer and laser diffraction measurements of the GO aqueous suspension (Figure S1) confirm this assumption, demonstrating that GO flakes have lateral size of up to 100 μm which complies with the highest values reported previously^43^.

After the GO reduction, the (00.2) XRD-pattern diffraction peak has shifted to higher angles due to the decrease in the rGO interlayer spacing, which proves elimination of the oxygen-containing groups. However, this peak positions are different for different types of the glass wafers. The interlayer spacing has been determined to be 4.1 Å for rGO_S-gl, 3.9 Å for rGO_AB-gl, and 4.5 Å for rGO-Mg-gl. These values are noticeably lower than the GO interlayer distance, which confirms elimination of interlayer water and oxygen-containing groups. On the other hand, these values are larger than both the graphite interlayer spacing of 3.4 Å and that of 3.7 Å published for the reduced graphene oxide^44^. This is due to retention of metal ions and carbonates formed during the reduction process, which cause an increase in the interlayer distance.

The diffraction patterns of the rGO_AB-gl and rGO_Mg-gl samples also contain distinguishable asymmetric (10.l) and (11.l) reflections which are superposition of reflections of the (hk.l) and (hk) types. The shapes and positions of these diffraction features coincide with those in microcrystalline graphite^45^ and suggest that an average lateral size of coherent scattering regions (CSR) corresponding to the defect-free regions in rGO_AB-gl and rGO_Mg-gl is 200 nm.

Figure 3b shows Raman spectrum of the initial GO with a broad G peak around 1595 cm^−1^ related to in-plane stretching of the graphene lattice and D peak around 1349 cm^−1^ caused by the lattice disorder, e.g., edges of the *sp*^2^ clusters and boundaries of the flakes^46,47^. After reduction, the frequency of the D band and G band in Raman spectra of all rGOs is equal to that of GO. The intensity ratio of these bands (I_D_/I_G_) is commonly used to evaluate the stacking order and defect density in the obtained graphene samples^12^. Upon the reduction, the I_D_/I_G_ ratio does not significantly change and remains within the range of 1.1, although commonly applied chemical reduction procedures lead to the significant rise of the I_D_/I_G_ ratio^39,48,49^. This evidences that the used reduction procedure does not cause considerable structural disorder if alkali-barium silicate glass or magnesium silicate glass are applied. The absence of the observable decrease in I_D_/I_G_ ratio is related to the high number of layers in the studied films that are about 500 nm in thick.

The absence of a high number of defects, e.g., nanosized holes and rips, which commonly arise due to removal of oxide groups^33,47^ in the obtained rGO_AB-gl and rGO_Mg-gl samples, is also shown by the obtained TEM images (Fig. 3e,f). The initial GO exhibits a continuous defect-free structure with the absence within GO flakes of any rips or holes with lateral size of more than tens of nanometers. The sharpness of the obtained diffraction spots and ratio between their intensities collectively demonstrate the monolayer character of the GO flakes. After the reduction with the alkali-barium silicate glass and magnesium silicate glass, no nanosized defects are observed in the structure of the rGO platelets (Fig. 3e,f). Moreover, a set of distinguishable hexagonal diffraction patterns rotated relative to each other can be observed in the case of rGO_Mg-gl (Fig. 3f (Inset)). This indicates that the obtained rGO_Mg-gl consists of the lamellar platelets combined in stacks of several layers having well-preserved crystalline structure with the long-range order of minimum several tens of nanometers. Note that this estimation coincides well with the aforementioned CSR area evaluated based on the X-ray diffraction data. In turn, the electron microdiffraction pattern of rGO_AB-gl (Fig. 3e (Inset)) is more ring-shaped (still having the six-fold graphene symmetry). This may be caused by high density of nanowrinkles arising due to the aforementioned retention of metal carbonates on the surface of rGO platelets.

The crumpled structure of the rGO platelet is also observed for the rGO_S-gl sample where sodium-containing species have been retained on the layer surfaces. However, in opposite to rGO_AB-gl and rGO_Mg-gl samples, rGO_S-gl exhibits quite defective structure. TEM image of this sample (Fig. 3d) demonstrates that large number of holes 5–10 nm in lateral size distributed within the structure of the layer. The low structural quality of the rGO_S-gl sample is also evidenced by low intensities of (10.l) and (11.l) reflections in the XRD pattern, although the I_D_/I_G_ ratio for rGO_S-gl is comparable to that for rGO_AB-gl. This discrepancy may be related to the sufficiently large distances between the formed holes and aforementioned large lateral size of the GO platelets, since the overall length of boundaries strongly affecting the intensity of the D band is considerably small in this case.

### Conductivity measurements

The difference in the efficiency of the GO reduction using different types of glass wafers is further evidenced by the conductivity measurements. The values of sheet resistance and corresponding conductivity values are summarized in Supplementary Table S1. The rGO_Mg-gl sample exhibits the highest conductivity of 33000 S*cm^−1^, whereas the conductivities of rGO_AB-gl and rGO_S-gl have been determined to be 10500 S*cm^−1^ and 117 S*cm^−1^, respectively. The values obtained for rGO_Mg-gl and rGO_AB-gl are comparable to those of rGO reduced by using borohydrides^49^, metal-acid solutions in the mild conditions^19^, and high-temperature annealing^50^. At the same time, rGO prepared using sodium silicate glass exhibits quite low conductivity due to highly defective nature of the rGO platelets.

### Reduction of the prior-formed GO films

One of the main issues that limits the use of the liquid-media chemical reduction method for preparing graphene for its further use is restacking of the suspension rGO platelets into graphite-like multilayer aggregates^48,51^. To solve this problem, the liquid-phase reduction of GO is carried out in strong basic or acidic solutions where the electrostatic repulsion of the remained functional groups prevents restacking^52^. Another approach is to modify rGO with various surfactants^53^.

However, the use of reducing agents that efficiently convert GO to rGO under mild conditions gives the opportunity to straightforwardly reduce not GO suspensions but GO films formed on various substrates prior to the reduction process. To analyze whether the method under consideration is applicable for effective reduction of GO films without their disruption, an additional series of experiments was performed. Particularly, GO films on quartz and silicon substrates were placed in aqueous media containing magnesium silicate glass wafer with its subsequent heating at 80 °C during 5 hours. Further characterization of the obtained sample by the means of UV-Vis and FTIR spectroscopy (Supplementary Figure S2), as well as elemental analysis (Supplementary Figure S3), indicates that the treated GO films were successfully converted to rGO with a high degree of reduction. This assumption is further confirmed by the conductivity measurements demonstrating that conductivity of the obtained reduced graphene oxide film is about 30000 S*cm^−1^.

**Figure 4:**
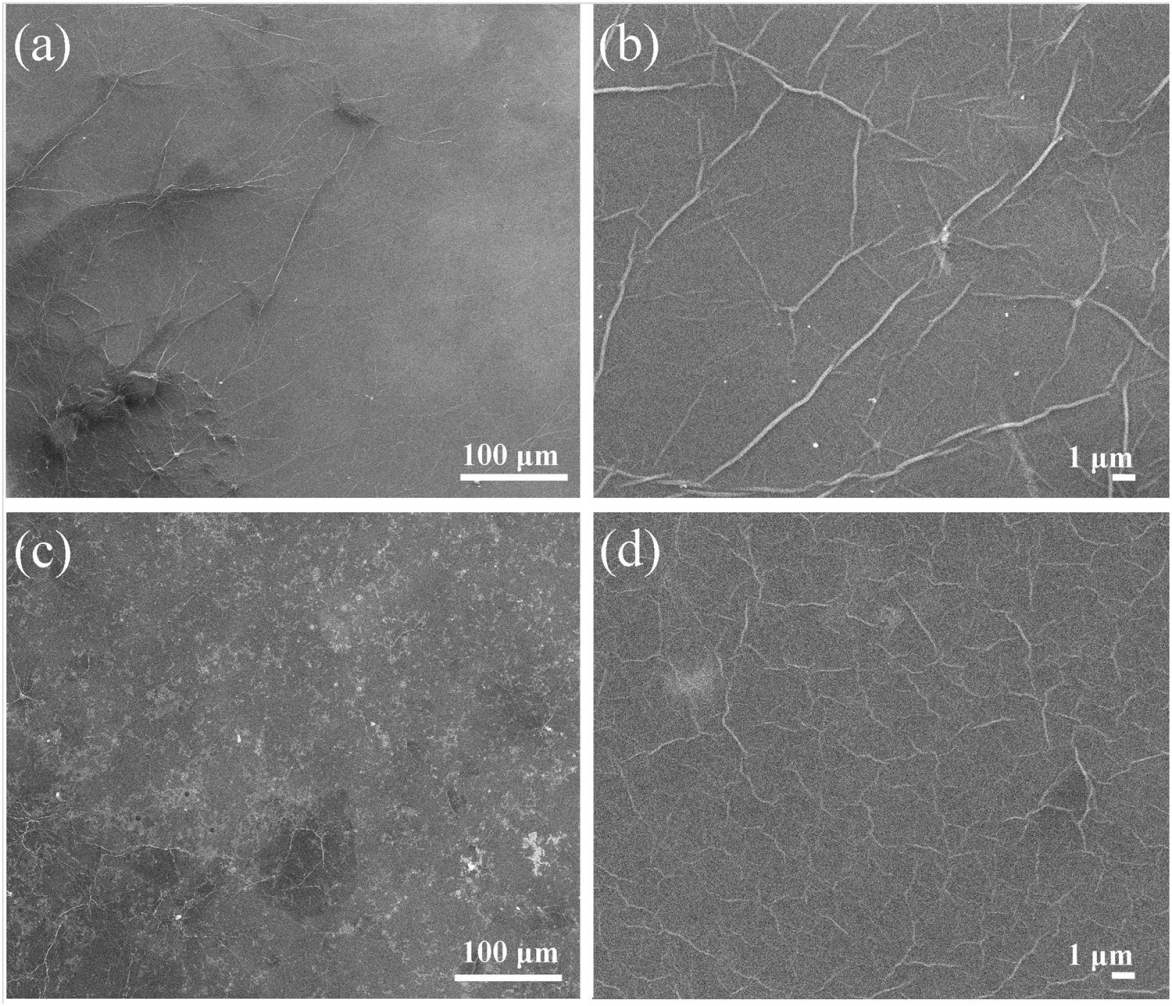
SEM images of the GO film (**a**,**b**) prior to and (**c**,**d**) after the reduction using magnesium silicate glass. The images demonstrate the absence of disruption of the film after the treatment.

Importantly, the applied reduction procedure also does not result in peeling of the GO film (Supplementary Figure S4) and formation of any observable defects, e.g., rips and tears, as is indicated by the obtained SEM images (Fig. 4). Note that bright areas observed in the SEM images of the rGO film originate from the oxidation of the silicon wafer surface (Figure S5). Thus, the considered reduction procedure can be applied to reduce not only the GO suspension but also GO films formed on the surface of various substrates that are widely used in graphene-based optoelectronic devices.

### Recyclability of the applied reducing agent

**Figure 5:**
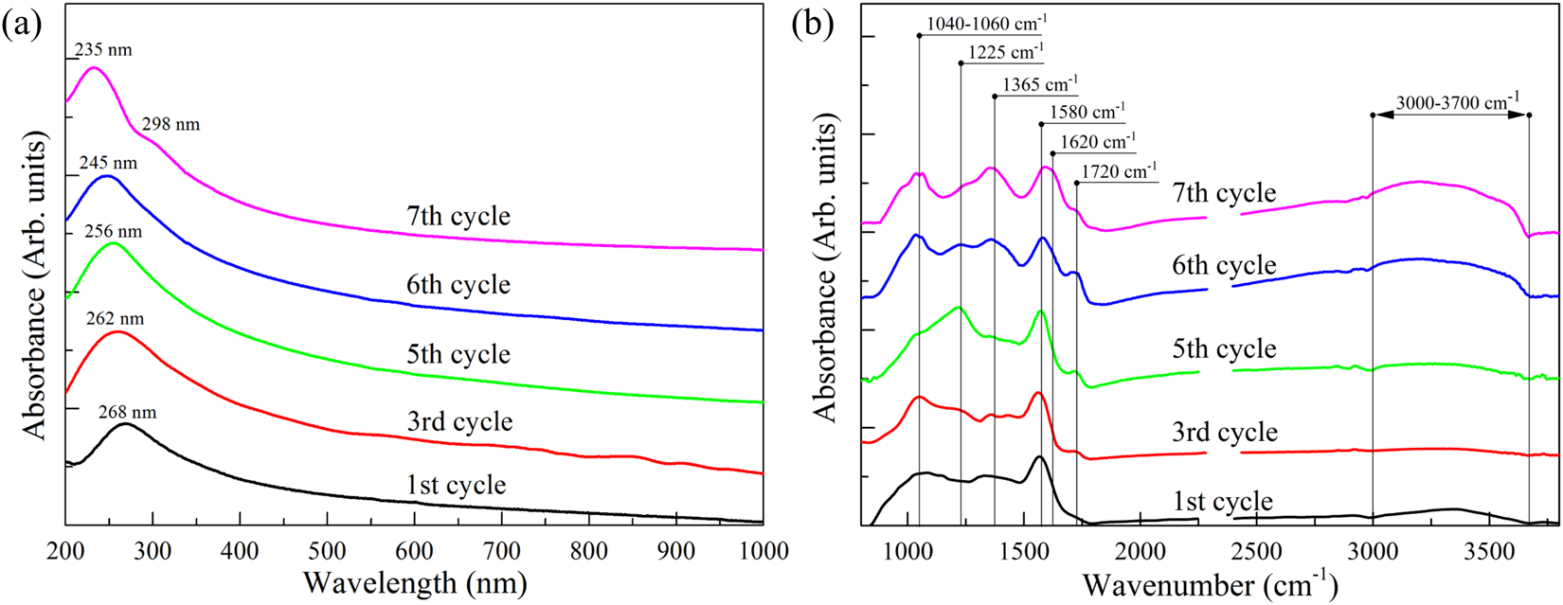
(**a**) UV-Vis and (**b**) FTIR spectra of GO deoxygenated with the reused magnesium silicate glass wafer. The obtained spectra demonstrate that glass wafers as a reducing agent can be reused up to five times without significant loss in the effectiveness.

The efficiency of using glass wafers as a reducing agent in converting GO into rGO further improved by their recyclability and simplicity of use. While conventional reducing agents are usually completely consumed during the reduction process, the glass wafer can be simply withdrawn from the aqueous media, washed by deionized water, and used again. The obtained UV-Vis and FTIR spectra (Fig. 5) demonstrate that a single glass wafer may be reused 5 times, providing effective reduction of GO. After the 5th cycle the reduction efficiency begins drastically decrease, and after the 7th cycle no significant elimination of the oxygen functionalities occurs. This is indicated by the shape of the corresponding UV-Vis spectrum (Fig. 5a, magenta curve) and retention of the distinguishable peaks at 1225 cm^−1^, 1365 cm^−1^ and 1720 cm^−1^ corresponding to basal-plane and edge-located oxygen-containing groups in the FTIR spectrum (Fig. 5b). Nevertheless, glass wafers are highly recyclable as GO-reducing agents and are much easier to be reused than other deoxygenating agents.

## Discussion

**Figure 6:**
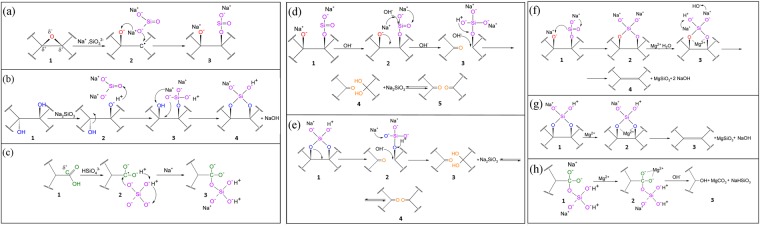
Illustration of the formation of intermediates consisting of the silicate anion (denoted by purple) and (**a**) epoxide (denoted by red), (**b**) hydroxyl (denoted by blue) or (**c**) carboxyl groups (denoted by green). (**d**,**e**) Elimination of the formed intermediates in the case of alkali silicate glass, resulting in removal of oxygen-containing groups with simultaneous perforation of the GO structure and formation of new carbonyl groups. (**f**–**h**) Removal of the intermediate formed by epoxide, hydroxyl and carboxyl groups in the presence of alkaline-earth cations.

The observed conversion of GO into rGO both by low-temperature heating in the presence of a glass wafer can be explained by the following mechanism. In heating a GO suspension with a glass wafer immersed into it to 60–80 °C, the wafer begins dissolving due to acid-catalyzed bimolecular displacement reactions^54–56^. This process results in leaching of the alkali and alkaline-earth cations (Na^+^, Mg^2+^, Ba^2+^, Ca^2+^) along with metasilicate (SiO3^2−^) and ortosilicate (SiO4^4−^) anions from the glass surfaces into the suspension^57–59^. In the process, the pH value rises from 3.4 to 8–8.5. Beside the formation of alkali and alkaline-earth silicates, the leached silicate anions may also interact with the oxygen-containing functional groups of GO. In this case intermediates are formed, composed by the metasilicate or ortosilicate anion that is attached to the GO layer by the oxygen-bridge bond originated from rearrangement of chemical bonds in epoxides (Fig. 6a), hydroxyls (Fig. 6b) or carboxyls (Fig. 6c). The assembled intermediates can be further eliminated by two possible ways depending on the chemical composition of the used glass wafer. If the alkali silicate glass wafer is used then further redistribution of the electron density in the intermediate may lead to the subsequent removal of the silicate anion. This results in the cleavage of carbon bond in the graphene network with formation of the carbonyl group (Fig. 6d,e). This way for eliminating oxygen functionalities from GO in the presence of alkali silicate glass is well supported by the observed rise in the carbonyl group concentrations and perforation of the carbon network in the rGO_S-gl sample.

In turn, the use of glass wafer that contains alkaline-earth metals, such as Mg or Ba, provides an alternative way of the intermediate transformation. In this case, an alkali-earth cation existing in the suspension interacts with the formed intermediate, resulting in their removal from GO with formation of the alkaline-earth metasilicate and alkali hydroxydes (Fig. 6f,g), while the graphene network remains intact. Furthermore, the presence of the alkaline earth cation also provides elimination of the carboxyl and carbonyl groups. In the case of carboxyls, the intermediate formed from the carboxyl groups and ortosilicate anion is removed and the carbonate along with phenol group are formed (Fig. 6h). Note that the supposed increase in the number of phenols groups after the reduction clearly manifests itself in arising of the 533.6-eV component in the O 1 s XPS spectra (Fig. 2d) of the rGO_AB-gl and rGO_Mg-gl samples. Elimination of carbonyls does not require silicate anions and is based on hydration of carbonyl group^60^ with formation of two adjusting hydroxyl groups. These groups further interact with the alkaline earth cation, resulting in their elimination and formation of alkaline earth hydroxide. Magnesium exhibit the lowest value of heat of hydroxide formation in the set of Mg, Ca and Ba^61^. This results in more effective removal of carbonyls using magnesium silicate glass then by using alkali-barium silicate glass, as is indicated by XPS data (Table 1). Moreover, Mg cation diffuse out more easily then Ba or Ca cation due to the lower ionic radius of the former one^62^. As a net result, magnesium silicate glass provide more effective reduction of GO.

Taking into account the data of the relative concentration of oxygen-containing groups in the initial GO and rGOs we further estimated the number of alkali-, alkali earth- cations and silicate anions consumed for reduction of the studied suspensions. Details of the calculations can be found in Supplementary materials and the obtained results are presented in Supplementary Tables S3 and S4. As seen, for the 200 µl of GO suspension 0.003 wt% in concentration the number of alkali and alkaline earth cations and silicate anions for all the used glass wafers lies within the range of 0.16 µmol, 0.085 µmol and 0.08 µmol, respectively. These values consist with the published data on the concentration of the anions and cations leached during the glass dissolving with the comparable mass and surface area^55,57^. The number of cations and anions required for conversion of 1 mg of GO into graphene was additionally calculated and the obtained values can be found in Supplementary Table S5.

To validate the proposed mechanism, a series of control experiments was carried out. In these experiments GO was heated under otherwise identical conditions in the presence of either a quartz wafer (rGO_Quartz), or magnesium sulphate (rGO_MgSO4), or sodium hydroxide (rGO_NaOH), or sodium silicate (rGO_Sil), or a combination of sodium silicate and magnesium sulphate (rGO_Sil-Mg). The degree of reduction and chemical composition determined for the resulting rGOs is indicative of the role of each component (silicate anions, alkali and alkaline-silicate cations) in reducing GO with glass wafers. The low-temperature (80 °C) heating of the GO aqueous suspension in the presence of the quartz wafer does not result in the deoxygenation of graphene oxide, as shown by UV-Vis, FTIR and XPS spectra (Figure S6). The treatment of GO with magnesium sulphate results in some elimination of the oxygen containing groups (Figure S7), but the reduction degree (C/O = 1.47) is drastically low and is not comparable to the values for the rGOs obtained using glass wafers. These results collectively indicate that introduction of both silicate anions and metal cations into the medium as a result of glass dissolving is of paramount importance for the GO reduction.

**Figure 7:**
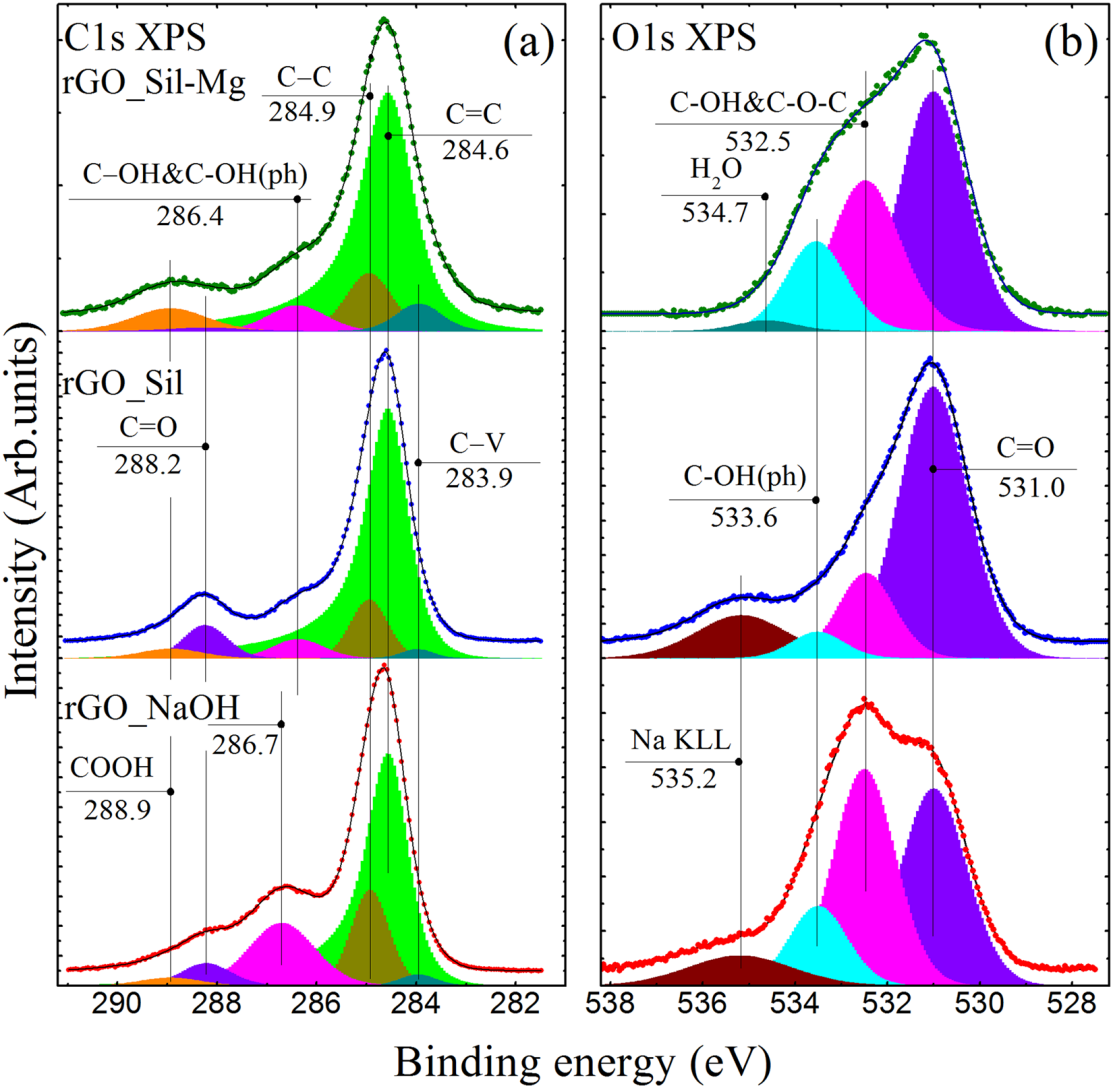
(**a**) High-resolution C1s and (**b**) O1s XPS spectra of initial GO reduced by alkaline solution (rGO_NaOH), sodium silicate (rGO_Sil), and sodium silicate with magnesium sulphate (rGO_Sil-Mg). The spectra and their fits are shown after the Shirley background subtraction and vertical offset from the fitting components.

The primary role of the silicate anions in the observed reduction process was further verified by analyzing the additional rGO_NaOH, rGO_Sil and rGO_Sil-Mg samples. Figure 7 demonstrates C1s and O1s XPS spectra of the obtained rGO samples and results of quantitative analysis of these XPS spectra presented in Table S2. Although strong alkaline solution has been reported to deoxygenate exfoliated GO sheets at the temperatures above 55 °C^36,39^, the rGO obtained in alkaline solution with pH~8 has the C/O ratio of 2.9 which is significantly lower than those even in the rGO_S-gl samples. On the other hand, the reduction degree of rGO_Sil (C/O = 4.99) is very close to that in rGO_S-gl (C/O = 4.2), and an increase in concentration of carbonyl groups is observed upon the reduction with both sodium silicate glass and sodium silicate powder. Moreover, the formation of nanoscale holes in the rGO_Sil sample is also demonstrated by the obtained TEM image (Figure S8). In turn, the rGO samples obtained either in the presence of glass wafers containing alkaline-earth oxides or by using sodium silicate mixed with magnesium sulphate exhibit almost equal C/O ratios and compositions of the residual groups. Thus, the obtained experimental results confirm the assessment that the studied reduction process originates from the presence of silicate anions. At the same time, the structural parameters and chemical composition of the obtained rGOs are determined by the type of the metal cations, presented in the suspension.

In summary, we for the first time have demonstrated that GO can be easily reduced by low-temperature heating in the presence of various glass wafers, namely, sodium silicate, magnesium silicate and alkaline-barium silicate glass wafers. The discussed method can be used to reduce both GO suspensions and GO films formed on various substrates without any considerable effect on the film morphology in the latter case. The additional studies have also confirmed recyclability of the glass wafers used as reducing agents, i.e., the possibility to efficiently reduce GO five times with a single glass wafer. The mechanism of the observed reduction process has been studied as well, revealing that the GO to rGO conversion by using glass wafers occurs due to cooperative interaction of the leached silicate anions and metal cations with oxygen-containing function groups of GO. The advantages of the proposed reduction method, i.e., its simplicity, low reaction temperature, recyclability and non-toxicity of the reducing agent, and the absence of strong acids and bases, make it attractive for the large-scale production of graphene and graphene-based materials for various applications, e.g., fabrication of composite fillers, graphene-based inks, and graphene coatings for optoelectronic devices.

## Methods

### Formation of GO suspensions and films

Graphene oxide was synthesized by the Hummers method^63^. In brief, graphite powder (4 g) was oxidized by using concentrated H_2_SO_4_, KMnO_4_, NaNO_3_, and H_2_O_2_ solutions. The resulting mixture was centrifuged (3500 rpm for 1 hour), and the supernatant was decanted away. The material remaining after this was additionally centrifuged (1500 rpm for 10 min) to obtain aqueous GO suspension as a supernatant. In the process of synthesis, sonication was excluded to prevent damaging of graphene oxide flakes and obtain suspensions with the utmost size of GO flakes (with lateral size of up to 100 μm).

To prepare GO films for the subsequent reduction, 200 μL of GO aqueous suspension 0.003 wt % in concentration was drop-casted on silicon and quartz wafers and dried overnight at room temperature.

### Reduction of GO

Three types of glass wafers with different chemical compositions were used as possible reducing agents for GO deoxygenation: sodium silicate glass containing only sodium oxide, magnesium silicate glass containing only sodium oxide and magnesium oxide, and alkali-barium silicate glass, as one of the most common glass types, containing various alkali and alkaline earth oxides. The chemical compositions of the used glass wafers and their price are presented in Table S6.

The reduction of GO aqueous suspensions was performed as follows: a piece of sodium silicate glass (10 × 6 mm wafer, 0.135 g), alkali-barium glass (16 × 7 mm wafer, 0.53 g) or magnesium silicate glass (14 × 8 mm wafer, 0.45 g) was immersed into GO aqueous suspension (40 mL) 0.01 wt% in concentration with subsequent stirring of the suspension at 80 °C for 5 hours in a fluoroplastic flask. The obtained rGO suspensions were copiously washed by centrifuging (centrifuge Sigma 3–30KS) at 26,200 rpm (60.600 g) and rinsing the obtained sediment with de-ionized water. The described purification procedure was repeated five times. The obtained rGO samples were denoted as rGO_S-gl (reduced by sodium silicate glass), rGO_AB-gl (reduced by alkali-barium silicate glass) and rGO_Mg-gl (reduced by magnesium silicate glass). The quantity of graphene, obtained from the 0.5 g piece of glass wafer (Sodium, Alkali Barium or Magnesium Silicate) with actual size of 15 × 7.5 × 1.0 mm was determined to be about 50 mg. Magnesium silicate glass wafer was applied for the reduction of graphene oxide suspension up to 7 times with successful conversion of GO into rGO during 5 cycles. As a result, the maximum quantity of the produced graphene from a single glass wafer in the applied conditions was determined to be about 0.25 g.

To analyze applicability of the studied method for reducing GO films on substrates, the GO films on quartz or silicon substrates were put into a fluoroplastic flask filled with de-ionized water (40 mL); after that, a piece of magnesium silicate glass was added, and the flask was heated at 80 °C for 5 hours. After the reduction, substrates with the rGO film were carefully withdrawn from the solution, washed several times with de-ionized water, and dried overnight at room temperature.

For better understanding of the processes that lie behind the observed deoxygenation of GO, a series of control experiments was carried out. Namely, GO aqueous suspensions were heated at 80 °C during 5 hours in the presence of the quartz wafer, after adding 0.01 mol. of magnesium sulphate powder (obtained from Acros Organics Company), or 150 µL of NaOH solution (0.1 M, obtained from Acros Organics Company), or 0.7 mmol. of sodium silicate powder (obtained from Acros Organics Company), or 0.8 mmol. of sodium silicate together with 0.8 mmol. of magnesium sulphate. The obtained samples were washed according to the aforementioned procedure.

To provide the correct alignment and deconvolution of the XPS spectra of GO and whole series of the studied rGOs, an additional rGO sample denoted as rGO_HT was prepared by annealing the GO film at 600 °C during 2 hours.

### Characterization of the obtained rGO samples

The pH values of the solutions were determined with a Fisher Scientific Accumet Basic AB15 pH meter. The UV-vis absorption spectra of the GO and rGO samples were collected with a Shimadzu-2450 spectrophotometer. Fourier transform infrared spectroscopy was performed on the Infralum-08 FTIR spectrometer equipped with the attenuation of total reflectance attachment. X-ray photoelectron spectroscopy (XPS) measurements were carried out on a Thermo Fisher ESCALAB 250Xi XPS system with a monochromatic Al Kα X-ray source (1486.6 eV). The spectra were calibrated with respect to the Au 4f7/2 line (84.0 eV). A surface charging revealed for low-conducting GO (and some rGOs) was taken into account by the aligning their XPS spectra with respect to the C1s line position (284.6 eV) for a well-conductive rGO-HT sample (see Supplementary Figure S9). The quantification and curve fitting of the obtained XPS spectra were performed by using standard CasaXPS software.

The X-ray diffraction (XRD) analysis was carried out using a Bruker Smart Apex Duo installation with a CuKα source and Apex 2D detector. The sample for diffraction measurements was fixed with nitrocellulose lacquer at the end of a cactus needle. Diffraction patterns were measured at various angles between the normal to the detector surface and the X-ray direction, the 2D data being subsequently recalculated to the 2θ configuration. The obtained diffraction patterns were analyzed using the DIFFRAC.EVA (Bruker Cor.) software based on the data from Powder Diffraction File ICCD PDF-2 release [JCPDS-International Centre for Diffraction Data (http://www.icdd.com)].

Raman spectra were obtained on a Horiba Jobin-Yvon LabRam HR800 installation equipped with a Laser Quantum Torus 532-nm laser 50 mW in output power. The exciting light was additionally attenuated with a filter having optical density of 1 and focused with a 20x objective lens into a spot approximately 30 μm in diameter. The power reaching the sample after passing the lightpath and objective was 0.11 mW.

Size distribution of GO and rGO flakes in aqueous solution was determined by laser diffraction measurements using Mastersizer 2000. Transmission electron microscopy (TEM) images were acquired with a Jeol JEM-2100F microscope (accelerating voltage 200 kV, point-to-point resolution 0.19 nm). Samples for TEM were prepared by deposition of aqueous GO and rGO suspensions 7·10−4 wt % in concentration onto conventional lacey carbon films. SEM images were collected with a JSM-7001F, Jeol microscope. Monolayer films for SEM imaging were prepared by the Langmuir−Blodgett method according to the procedures published elsewhere^64^. Surface morphology and thickness of the rGO films were analyzed with a Veeco Dimension 3100 atomic force microscope operating in the tapping mode by using RTESP probes.

Electrical conductivity measurements of the GO and rGO samples were performed on the base of two-electrode system. The GO and rGO films were deposited by the drop-casting method onto the surface of quartz substrates with two comb Au electrodes 80 nm thick separated by 500 µm. The electrode comb consisted of 8 pairs of the electrode bars (Figure S10).

## Electronic supplementary material

Supplementary Information
