# Supplementary material for: Facile reduction of graphene oxide suspensions and films using glass wafers
Source: Sci Rep. 2018 Sep 20;8:14154. doi: 10.1038/s41598-018-32488-x (PMC6147865; doi:10.1038/s41598-018-32488-x)
Supplement: Supplementary file 1 — Supplementary Information [file 41598_2018_32488_MOESM1_ESM.docx]

Supplementary information

# **Facile reduction of graphene oxide suspensions and films using glass wafers**

Maxim K. Rabchinskii,^1^ Arthur T. Dideikin,^1^ Demid A. Kirilenko,^1,2^* Marina V. Baidakova,^1,2^ Vladimir V. Shnitov,^1^ Friedrich Roth,^3^ Sergei V. Konyakhin,^1,4,5^ Nadezhda A. Besedina,^1,4^ Sergei I. Pavlov,^1^ Roman A. Kuricyn,^1^ Natalie M. Lebedeva,^1^ Pavel N. Brunkov^2^ and Alexander Ya. Vul’^1^

*^1^ Ioffe Institute, 26 Politekhnicheskaya, Saint-Petersburg 194021, Russia*

*^2^ ITMO University, 49 Kronverksky Pr., Saint-Petersburg 197101, Russia*

*^3^ TU Bergakademie Freiberg, Institute of Experimental Physics, Leipziger Straße 23, Freiberg D-09599, Germany*

*^4^ St. Petersburg Academic University, St. Petersburg 194021, Russia*

*^5^ Institute Pascal, PHOTON-N2, University Clermont Auvergne, CNRS, 4 avenue Blaise Pascal, 63178 Aubiere Cedex, France*

* - Corresponding author: Demid A. Kirilenko, E-mail address: Demid.Kirilenko@mail.ioffe.ru Tel: +7 905 226 29 13 Fax: +7 812 297 00 73


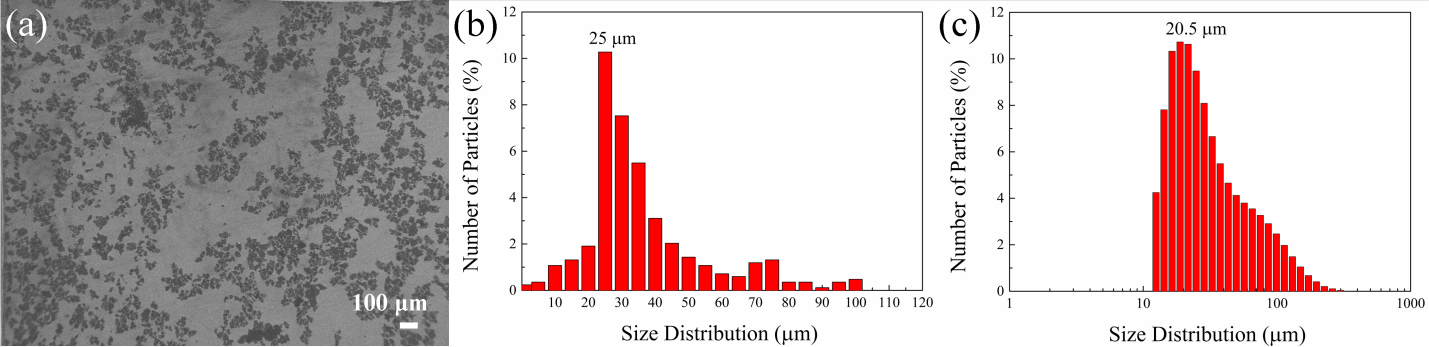


Figure S1 | (a) SEM image of GO flakes on the silicon substrate and size distributions obtained from (b) the analysis of SEM images and (c) using the Laser Diffraction method. As seen, GO flakes with the lateral size of up to 25 μm are mainly present in the used graphene oxide suspension, while the maximum lateral size exceeds 100 μm.

Table S1**.** Sheet resistance and the corresponding conductivity values of the obtained rGOs.

| Sample | Sheet resistance, Ω/sq | Conductivity, S*cm^-1^ |
| --- | --- | --- |
| GO | >10^12^ | - |
| rGO_S-gl | 7*10^8^ | 120 |
| rGO_AB-gl | 6*10^6^ | 10500 |
| rGO_Mg-gl | 2*10^5^ | 33000 |


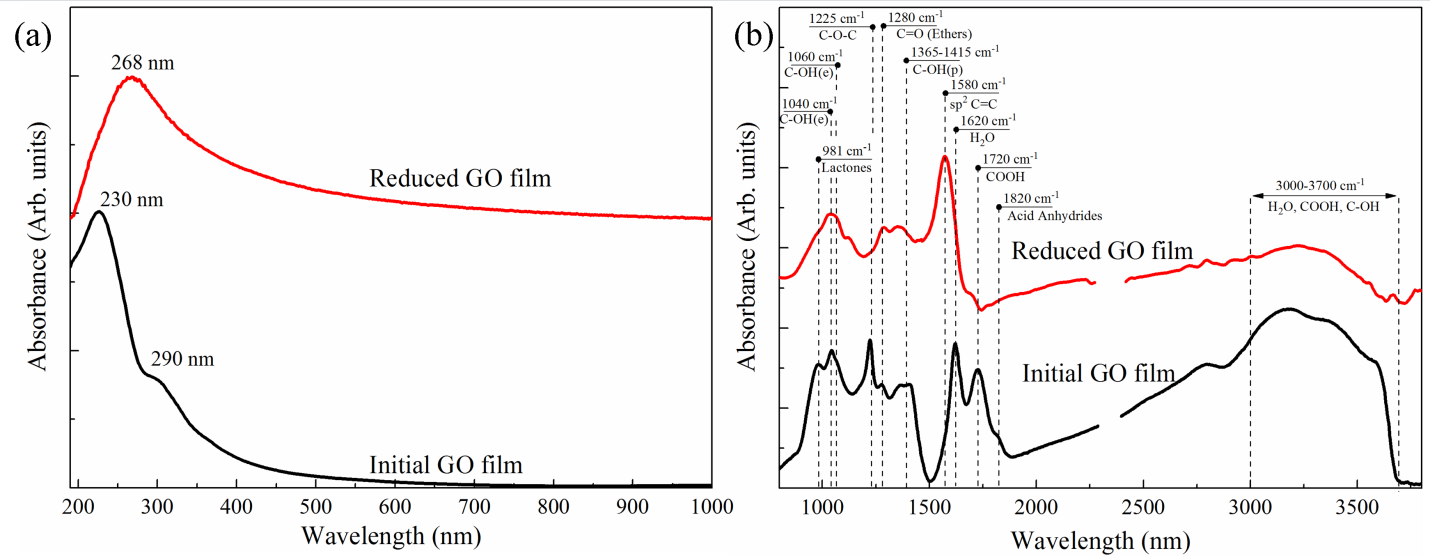


Figure S2 | (a) UV-Vis and (b) FTIR spectra of the GO film formed prior to the reduction process and deoxygenated by heating at 80 °C for 5 hours in aqueous media in the presence of magnesium silicate glass.


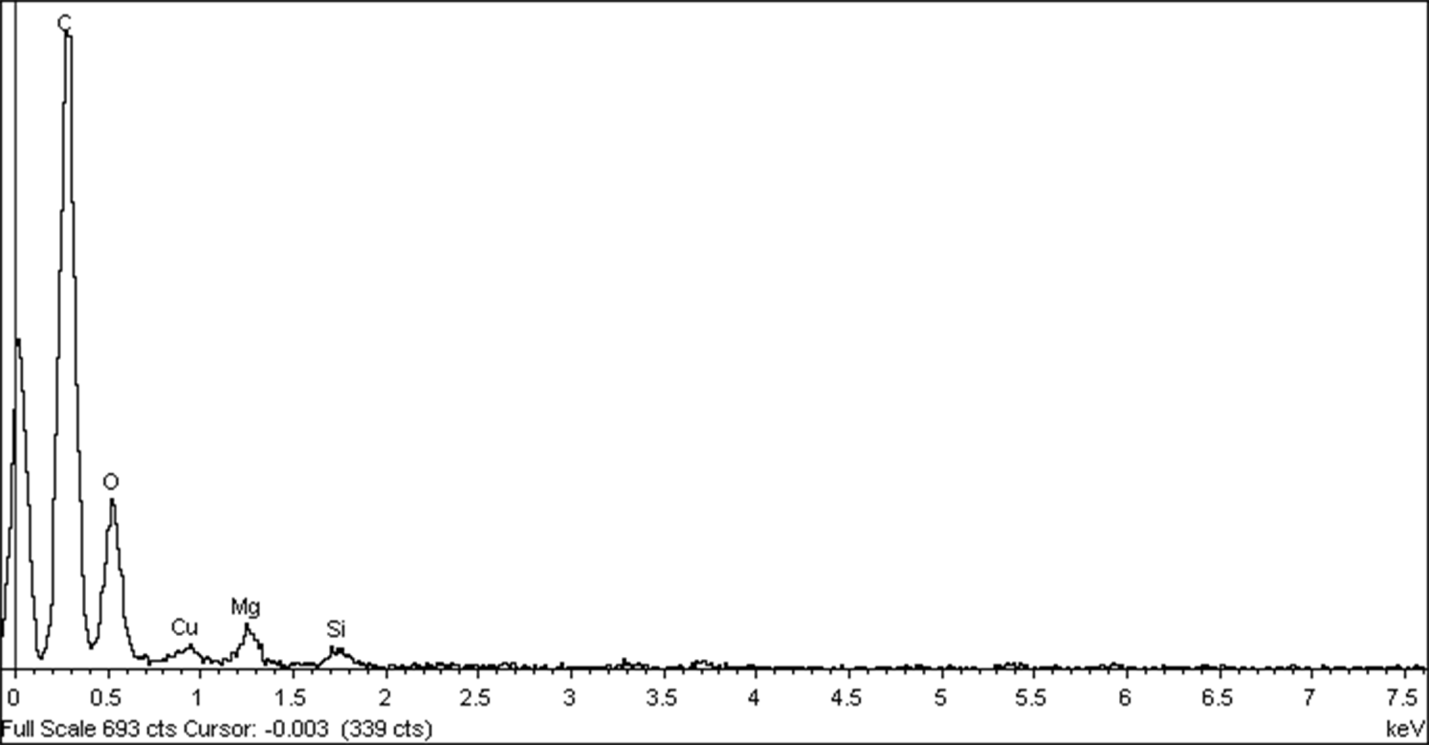


Figure S3 | X-ray microanalysis spectrum of the GO film reduced by using magnesium silicate glass.


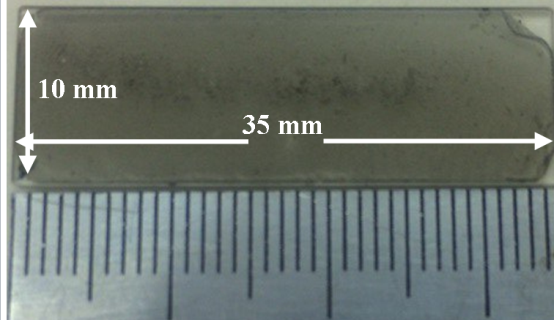


Figure S4 | Optical image of the GO film with the 35 x 10 mm lateral size reduced using magnesium silicate glass. As seen, the film remained intact after the reduction without peeling from the quartz substrate.


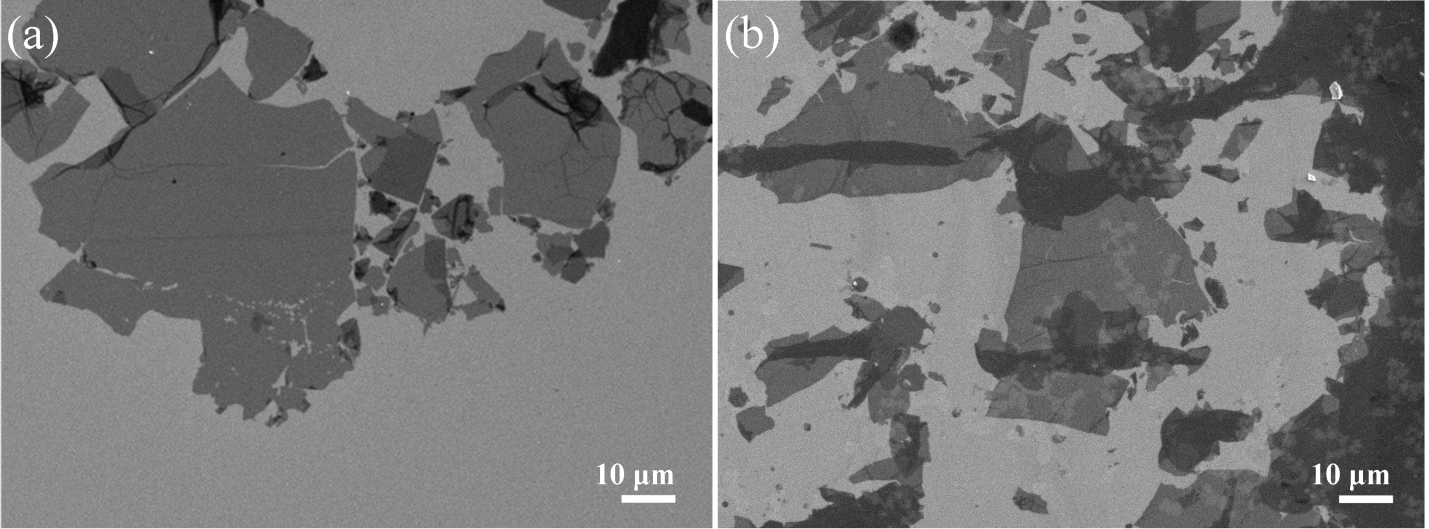


Figure S5 | SEM images of the monolayer GO platelets on the silicon surface prior to and after the reduction with magnesium silicate glass. Continuous brighter areas can be observed both under the monolayer platelet and on the free surface of the wafer, indicating that they are caused by the silicon substrate oxidation.


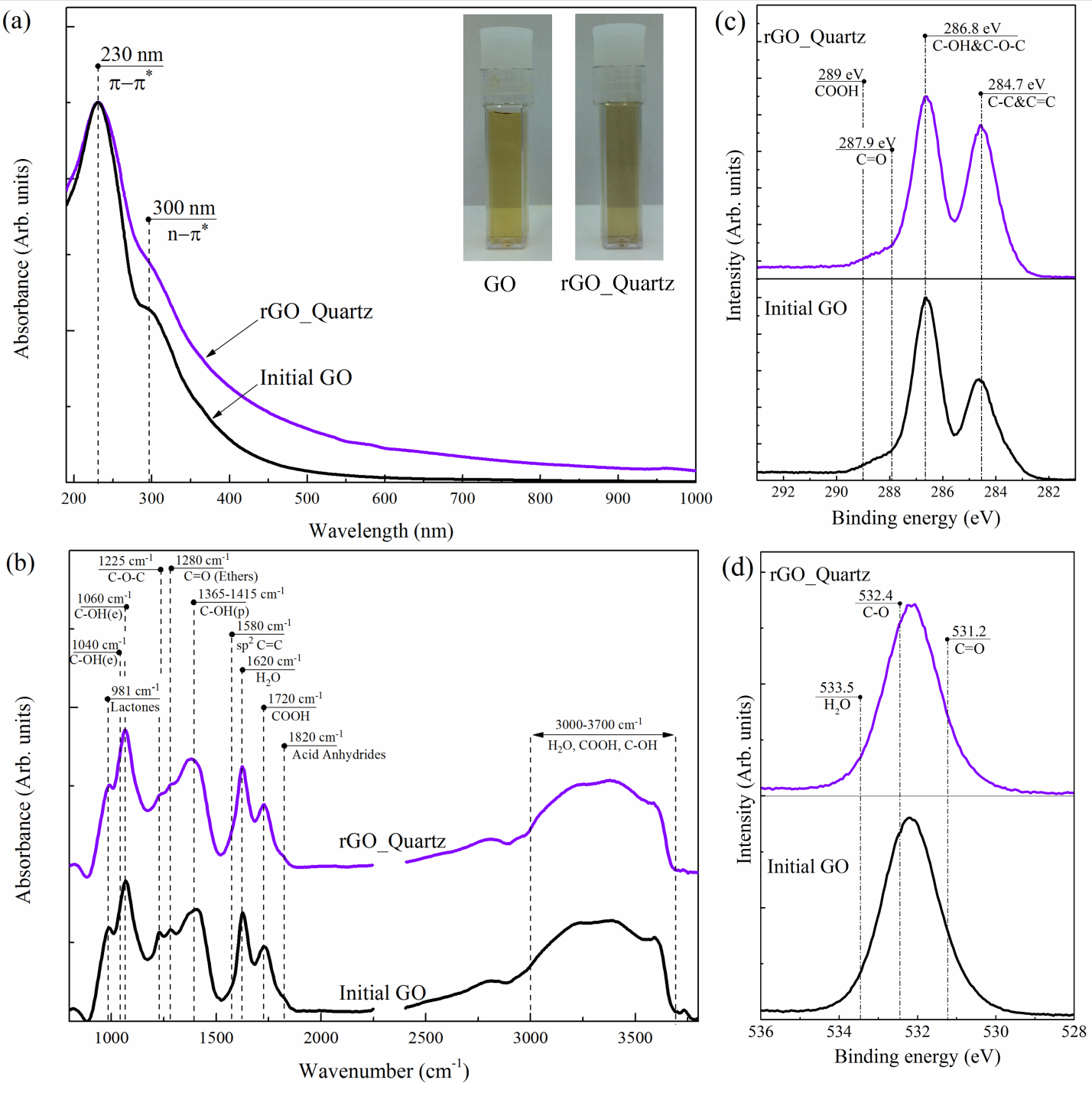


Figure S6 | (a) UV-Vis, (b) FTIR, (c) C1s and (d) O1s XPS spectra of the initial GO and GO suspension heated in the presence of the quartz wafer. The obtained results demonstrate that no effective deoxygenation of graphene oxide proceeds if GO aqueous suspension is heated at 80 °C in the presence of the quartz wafer.


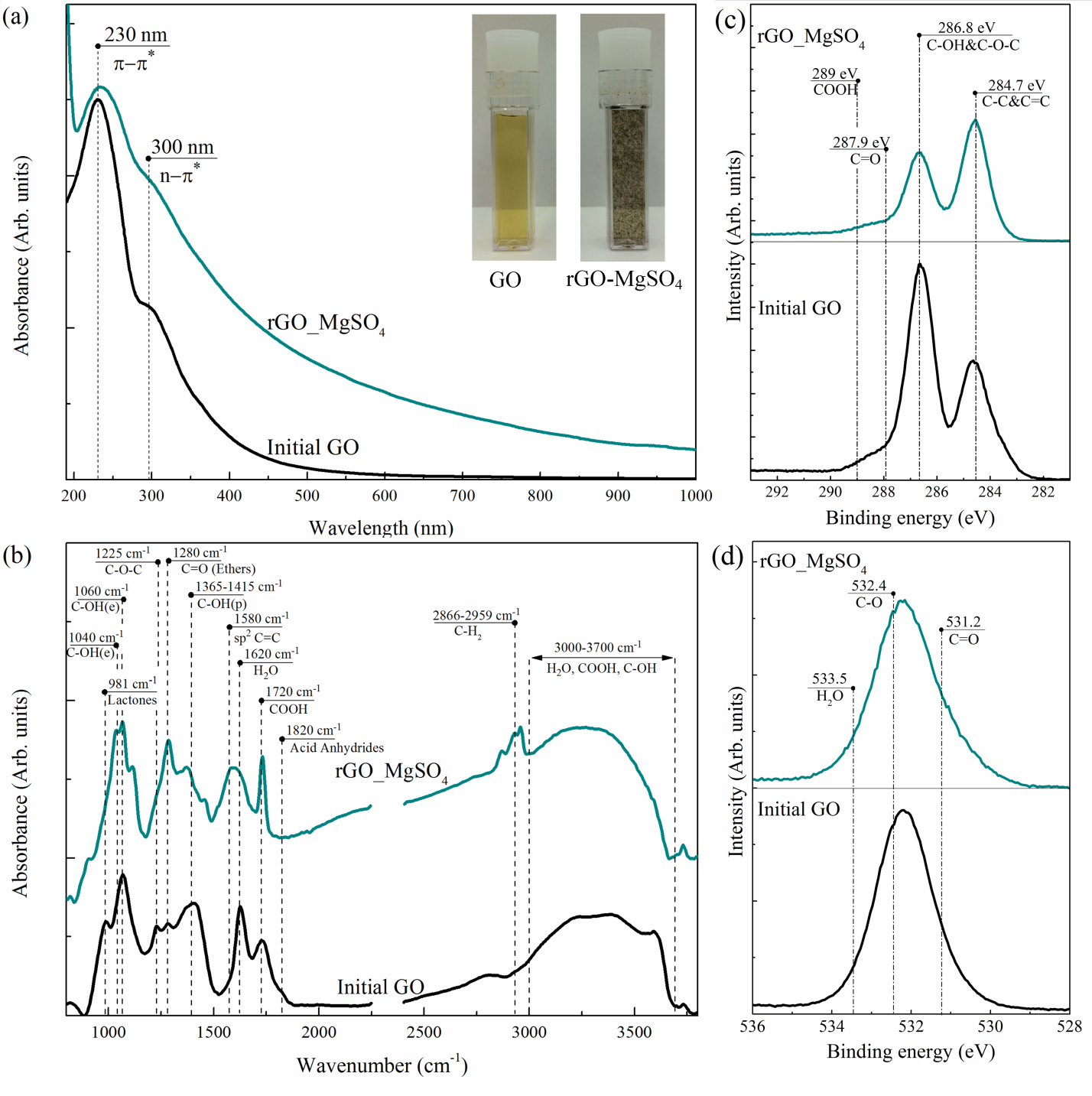


Figure S7 | (a) UV-Vis, (b) FTIR, (c) C1s and (d) O1s XPS spectra of the initial GO and GO suspension heated in the presence of 0.01 mol of MgSO_4_. As in the case of the quartz wafer, no significant graphene reduction is observed.

Table S2 | The C/O ratios and relative concentrations of functional groups determined by deconvolution of C1s XPS spectra for rGOs obtained using sodium hydroxide (rGO_NaOH), sodium silicate (rGO_Sil), and combination of sodium silicate and magnesium sulphate (rGO_Sil-Mg)

| Component | C-V | C=C | C-C | C-OH & C-O-C | >C=O | O=C-OH | C/O Ratio |
| --- | --- | --- | --- | --- | --- | --- | --- |
| Binding  Energy (eV) | 283.9 | 284.6 | 284.9 | 286.8 | 287.7 | 288.8 |  |
| rGO_NaOH | 0.018 | 0.570 | 0.157 | 0.182 | 0.049 | 0.022 | 2.92 |
| rGO_Sil | 0.018 | 0.716 | 0.112 | 0.052 | 0.079 | 0.023 | 4.99 |
| rGO_Sil-Mg | 0.049 | 0.658 | 0.131 | 0.083 | 0.023 | 0.056 | 5.39 |


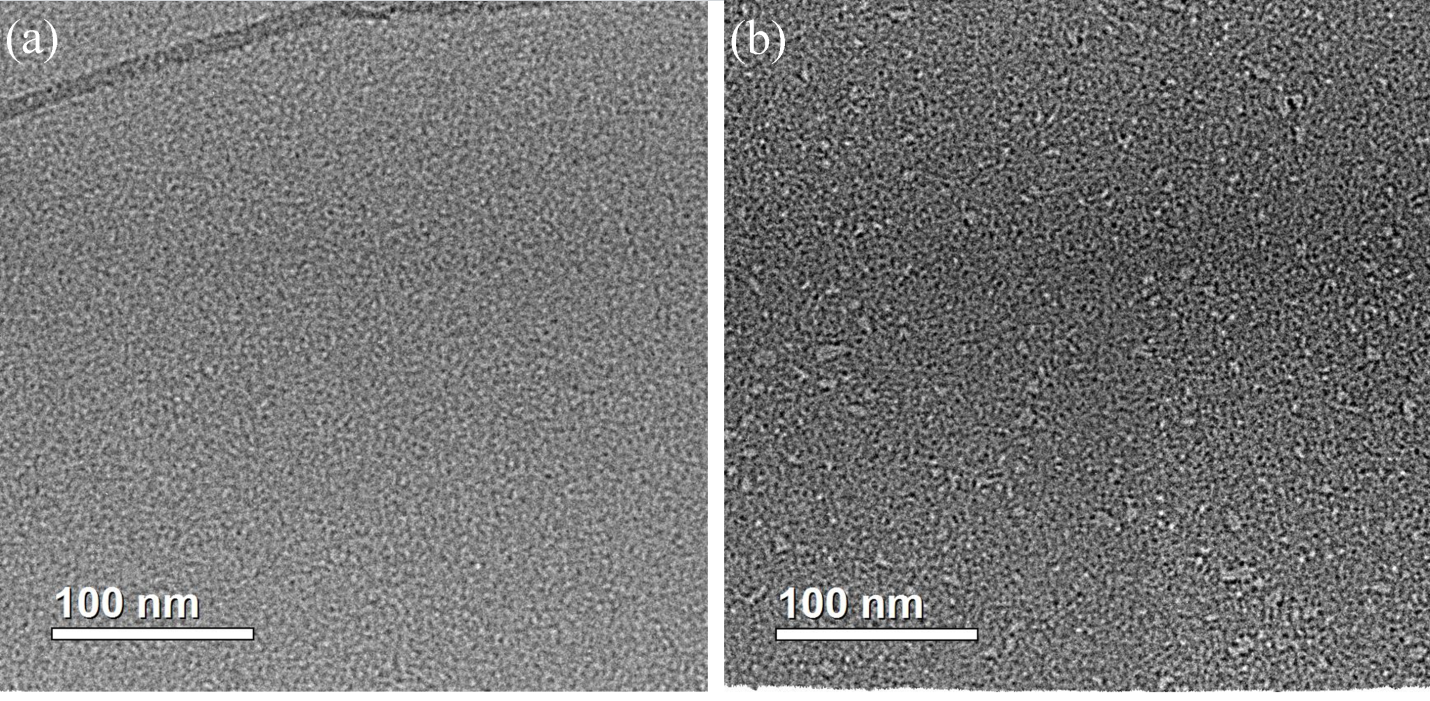


Figure S8 | TEM images of the (a) initial GO and (b) rGO-Sil sample. The images demonstrate formation of arrays of holes with lateral size of 5-20 nm after the reduction, as in the case of the rGO_S-gl sample.

*Calculation of the consumed cations and anions*

Let us first estimate the quantity of alkali-, alkali earth- cations and silicate anions consumed during the studied reduction of 200 ul 0.003 wt% (0.03 mg/ml) graphene oxide using sodium silicate glass, magnesium silicate glass and alkali-barium silicate glass. Firstly, the number of the eliminated basal-plane (C-OH and C-O-C), carbonyl (C=O) and carboxyl (COOH) functional groups must be calculated. This can be done using following expression:

$$N_{func.group}= N_{C}*\left[ D_{GO}^{func.group}-D_{rGO}^{func.group} \right]*N_{flakes} (1)$$

where N_c_ is mean number of carbon atoms in the single graphene oxide flake and N_flakes_ is the number of graphene oxide flakes in the studied suspension. The terms $D_{GO}^{func.group}$ and $D_{rGO}^{func.group}$ are the relative concentrations of the calculated type of functional group (either basal-plane or carbonyl or carboxyl) in the initial graphene oxide and the obtained reduced graphene oxide, respectively. These values are presented in the Table 1. Note that if relative concentration of the functional group is higher in the rGO sample than in the initial GO then no elimination of this group has proceeded during the reduction and $D_{GO}^{func.group}$ - $D_{rGO}^{func.group}$ = 0. The value of N_c_ can be derived as

$$N_{C}=\frac{S_{m}(Flake)}{S(Single hexagon)}*A*\frac{1}{3} (2)$$

where S_m_(Flake) is the mean surface area of graphene oxide flakes, S(Single hexagon) is the area of hexagonal unit in the graphene oxide lattice and A = 6 is the number of carbon atoms in one hexagon. The term 1/3 is related to the fact that every single carbon atom is a part of three adjacent hexagon in graphene lattice. Considering the fact that mean diameter of the graphene oxide flakes in the studied suspensions is 25 µm (See Figure S1) and the length of carbon bonds in the graphene oxide network is about 1.48 Å, one can roughly estimate the number of carbon atoms in a single GO flake

*N_C_ =* 1.75*10^10^

At the same time, number of GO flakes can be determined as

$$N_{flakes}=\frac{m_{GO}}{m_{flake}} (3)$$

where $m_{GO}$ is the total mass of GO in the suspension and $m_{flake}$ is the mass of a single graphene oxide flake. The former one can be estimated from the following relation

*m(GO) = V_(GO)_ * C_(GO)_* (4)

Since V*_(GO)_ = 0.2* ml and *C_(GO)_* = 0.03 mg/ml, the total mass of GO is determined to be 6*10^-6^ g. The mass of a single GO flake can be expressed as

$$m\left( GO flake \right)=N_{C}*m\left( C \right)+N_{C}*\left[ \left( D_{GO}^{BasalPlane}\left( A*\left. \left\{ m\left( O \right)+m\left( H \right) \right\} \right.+ m\left( O \right) \right) \right)+D_{GO}^{C=O}*m\left( O \right)+ D_{GO}^{C=O}*\left( 2*m\left( O \right)+m\left( H \right)+m(C) \right) \right] (5)$$

where m(C), m(O) and m(H) are the masses of carbon, oxygen and hydrogen atoms in grams, respectively. The term A=1.5 is the relation between hydroxyl and epoxide groups from the modified Lerf Klinowski model. Using Eq (5) and considering that m(C) = 19,93*10^-24^, m(O) = 26,67*10^-24^ and m(H) = 1,674*10^-24^ one can estimate that

*m(GO flake) =* 1.0386*10^-12^ g

Thus, from Eq (3)

$$N_{flakes}=5.777*{10}^{6}$$

Using Eq (1), the calculated *N_C_*  and $N_{flakes}$ values, and $D_{GO}^{func.group}$ and $D_{rGO}^{func.group}$ from the Table 1 one can estimate the number of the oxygen functionalities eliminated from for the each type of the used glass. The obtained values are presented in Table S3.

Table S3 | Number of the eliminated oxygen-containing functional groups for the each rGO sample

| Oxygen-containing group | C-OH & C-O-C | >C=O | O=C-OH |
| --- | --- | --- | --- |
| rGO_S-gl | 4.387*10^16^ | 0 | 0 |
| rGO_AB-gl | 4.418*10^16^ | 0.303*10^16^ | 0.202*10^16^ |
| rGO_Mg-gl | 4.620*10^16^ | 0.172*10^16^ | 0.202*10^16^ |

Now we can determine the number of alkali, alkali-earth cations and silicate anions, consumed during the studied reduction processes. In the case of basal-plane groups, two sodium cations and one silicate anion are required for the formation of the intermediate and its elimination for all types of the glass wafers. At the same time, in the case of alkali-barium and magnesium silicate glass wafers one sodium cation and one silicate anion are consumed forming intermediate with carboxyl group. Moreover, additional sodium cation take part in the hydroxylation of carbonyl group. Considering this and the estimated number of the eliminated oxygen-containing functional groups presented in Table S3, the maximum number of consumed sodium cations and silicate anions are 0.1457 µmol (8.774*10^16^) and 0.0728 µmol (4.387*10^16^) in the case of sodium silicate glass, 0.1551 µmol (9.341*10^16^) and 0.0767 µmol (4.620*10^16^) in the case of alkali-barium silicate glass, 0.1596 µmol (9.614*10^16^) and 0.0801 µmol (4.822*10^16^) for magnesium silicate glass, respectively. Reduction of graphene oxide using alkali-barium and magnesium silicate glass also requires one alkali-earth cation for each type of group. Thus, the number of consumed alkali-earth cations is estimated to be 0.0817 µmol (4.923*10^16^) and 0.0829 µmol (4.994*10^16^) for alkali-barium and magnesium silicate glass wafers, respectively. For clearance, all of the obtained values are presented in Table S4.

It is worth mentioning that one of the final product of the reduction is Na_2_SiO_3_ that is easily dissociating to Na_2_O and SiO_2_ with the following formation of Na^+^ and SiO_3_^2-^ in acidic solutions. As a result, the number of the required sodium cations silicate anions is at least 3 orders lower than the estimated value, considering the Na_2_SiO_3_ dissociation rate.

Table S4 | Number of the consumed alkali, alkali-earth cations and silicate anions in the experimentally studied reduction processes

|  | Alkali cation | Alkaline earth cation | Silicate anion |
| --- | --- | --- | --- |
| rGO_S-gl | 0.1457 µmol  (8.774*10^16^) | 0 | 0.0728 µmol  (4.387*10^16^) |
| rGO_AB-gl | 0.1551 µmol  (9.341*10^16^) | 0.0817 µmol  (4.923*10^16^) | 0.0767 µmol  (4.620*10^16^) |
| rGO_Mg-gl | 0.1596 µmol (9.614*10^16^) | 0.0829 µmol  (4.994*10^16^) | 0.0801 µmol (4.822*10^16^) |

Finally, we can estimate the number and molar ratio of the alkali, alkali-earth cations and silicate anions that is required for the reduction of 1 mg of graphene oxide. This can be done simply by dividing the obtained values presented in Table S4 by the mass of the GO in the studied suspension, 6*10^-3^ mg. The obtained values are presented in Table S5.

Table S5 | Number of the consumed alkali, alkali-earth cations and silicate anions per 1 mg of graphene oxide

|  | Alkali cation | Alkali-earth cation | Silicate anion |
| --- | --- | --- | --- |
| rGO_S-gl | 24.283 µmol | 0 | 12.133 µmol |
| rGO_AB-gl | 25.851 µmol | 13.616 µmol | 12.783 µmol |
| rGO_Mg-gl | 26.6 µmol | 13.817 µmol | 13.35 µmol |

Table S6 | Chemical compositions of the applied glass wafers and price for the corresponding wafer with size of 35x10x1.0 mm and mass of 0.25 g, provided by Corning Inc. company

| Glass | Content/mol(%) | | | | | | | Price of the wafer, $ |
| --- | --- | --- | --- | --- | --- | --- | --- | --- |
|  | SiO_2_ | Al_2_O_3_ | Na_2_O | K_2_O | MgO | BaO | CaO |  |
| Sodium silicate glass | 88 | 0 | 12 | 0 | 0 | 0 | 0 | 0.2 |
| Magnesium silicate glass | 78 | 0 | 15 | 0 | 7 | 0 | 0 | 1.7 |
| Alkali-barium silicate glass | 74 | 0.8 | 8.1 | 5.6 | 1.5 | 6.3 | 3.7 | 0.6 |





Figure S9 | High-resolution (a) C1s and (b) O1s XPS spectra of the initial graphene oxide sample reduced by annealing at 600 °C for 2 hours. The spectra were used as references in aligning and interpreting all the XPS spectra of the GO and rGOs samples obtained in this work.


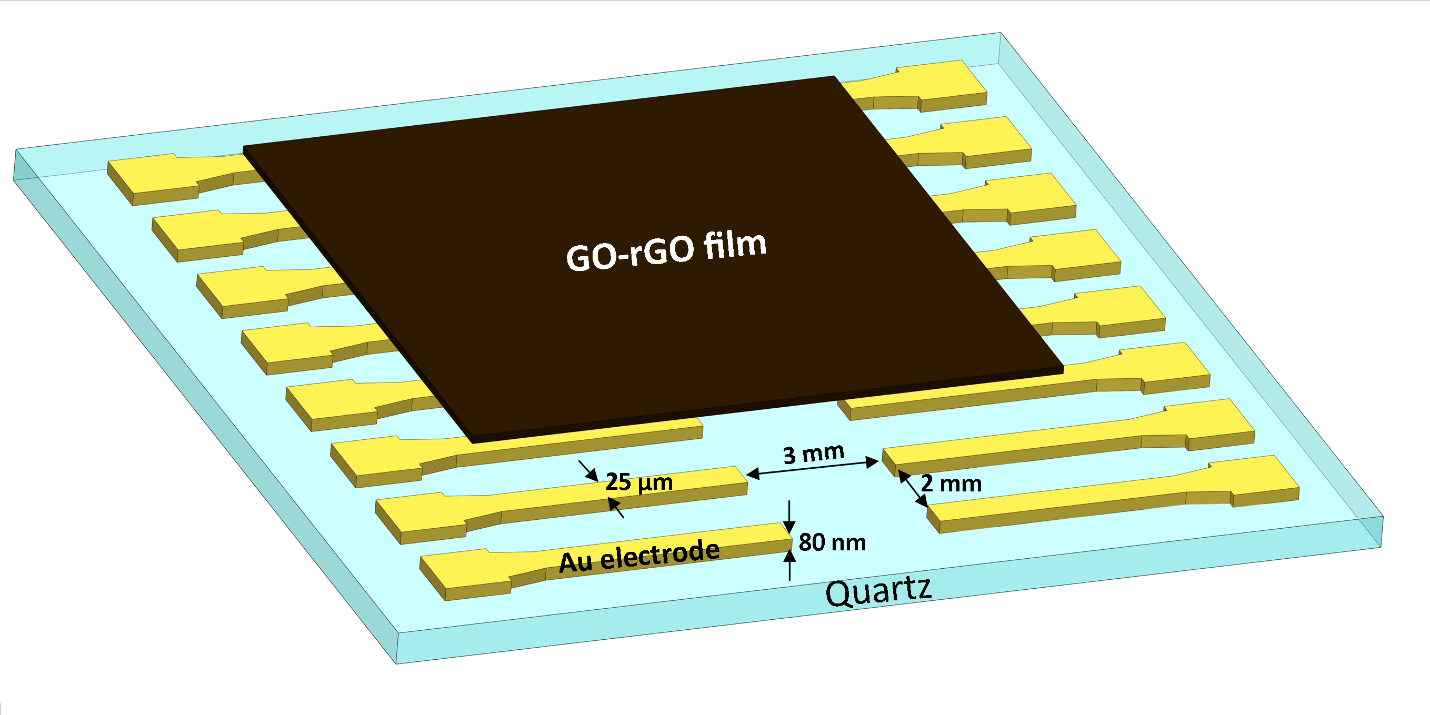


Figure S10 | The electrode substrate model for conductivity measurements of GO and rGO films. Not in scale.
